# Exploring NK‐Cell molecules that impact the immune response and microenvironment in head and neck squamous cell carcinoma

**DOI:** 10.1111/jcmm.18045

**Published:** 2023-11-27

**Authors:** Peng Shao, Wei‐Wei Hu, Xin‐lian Shi, Ming‐yang Shu, Dong‐Ya Li, Tingting Zhou, Qi‐Tao Zhao

**Affiliations:** ^1^ Department of Stomatology Huai 'an Second People's Hospital and The Affiliated Huai an Hospital of Xuzhou Medical University Huai 'an Jiangsu China

**Keywords:** HNSCC, immune microenvironment, immunotherapy, NK cell, prognosis

## Abstract

NK cells play a role in various cancers, but their role in head and neck squamous cell carcinoma (HNSCC) still needs to be explored. All public data are obtained from the Cancer Genome Atlas Program (TCGA) database. All analysis was performed using specific packages in R software. In our study, we quantified the immune microenvironment of HNSCC through multiple algorithms. Next, we identified NK cell‐associated genes by quantifying NK cells, including SSNA1, TRIR, PAXX, DPP7, WDR34, EZR, PHLDA1 and ELOVL1. Then, we explored the single‐cell expression pattern of these genes in the HNSCC microenvironment. Univariate Cox regression analysis indicated that the EZR, PHLDA1 and ELOVL1 were related to the prognosis of HNSCC patients. Following this, we selected EZR for further analysis. Our results showed that the patients with high EZR expression might have a poor prognosis and worse clinical features. Biological enrichment analysis showed that EZR is associated with many oncogenic pathways and a higher tumour stemness index. Meanwhile, we found that EZR can remodel the immune microenvironment of HNSCC. Moreover, we noticed that EZR could affect the immunotherapy and specific drug sensitivity, making it an underlying clinical target. In summary, our results can improve the understanding of NK cell in HNSCC. Meanwhile, we identified EZR as the underlying clinical target of HNSCC.

## 1 INTRODUCTION

Head and neck squamous cell carcinoma (HNSCC) is a malignant tumour originating from the mucosal epithelium of the head and neck, including the oral cavity, pharynx, larynx and other head and neck regions.^1^ HNSCC is one of the most common head and neck malignancies worldwide, and its morbidity and mortality are relatively high. Smoking, alcohol consumption, betel nut chewing and human papillomavirus (HPV) infection are major risk factors for HNSCC.^2^ Among them, the incidence of HNSCC, especially oropharyngeal cancer, associated with HPV infection has increased in recent years, especially in young populations.^3^ The treatment of HNSCC mainly includes surgery, radiotherapy and chemotherapy. In early‐stage HNSCC, surgery or radiotherapy is usually the treatment of choice. For advanced or recurrent diseases, comprehensive treatment strategies are more common.^4^ But even after comprehensive treatment, the five‐year survival rate of advanced HNSCC is still not high.^5^ In recent years, with an in‐depth understanding of tumour biology, targeted therapy and immunotherapy have gradually become research hotspots in the treatment of HNSCC.^6^ Some new therapeutic drugs, such as immune checkpoint inhibitors against PD‐1/PD‐L1, have shown promise in the treatment of HNSCC, bringing new treatment options for patients.^7^

NK cells, or natural killer cells, are a key component of the innate immune system. Such cells have a specific killing effect on non‐self and altered cells in the body, such as virus‐infected cells or tumour cells.^8^ Unlike T cells and B cells, NK cells do not need to be stimulated by specific antigens to display their cell‐killing activity.^9^ In cancer, NK cells play a crucial role. They can identify and destroy early tumour cells, thereby preventing further development and metastasis of tumours.^10^ However, many established tumours can evade NK cell surveillance and attack through various mechanisms, such as secreting immunosuppressive factors or changing their antigenic properties.^11^ Therefore, tumour immune escape from NK cells is a challenge in cancer therapy.^12^ In recent years, exploiting the antitumor activity of NK cells as a cancer treatment strategy has attracted considerable attention.^13^ For example, NK cell immunotherapy, which aims to enhance the ability of NK cells to attack tumours, is gradually becoming a promising treatment method.

In our study, we quantified the immune microenvironment of HNSCC through multiple algorithms. Next, we identified NK cell‐associated genes by quantifying NK cells, including SSNA1, TRIR, PAXX, DPP7, WDR34, EZR, PHLDA1 and ELOVL1. Then, we explored the single‐cell expression pattern of these genes in the HNSCC microenvironment. Univariate Cox regression analysis indicated that the EZR, PHLDA1 and ELOVL1 were related to the prognosis of HNSCC patients. Following this, we selected EZR for further analysis. Our results showed that the patients with high EZR expression might have a poor prognosis and worse clinical features. Biological relation analysis showed that EZR is associated with many oncogenic pathways and a higher tumour stemness index. Meanwhile, we found that EZR can remodel the immune microenvironment of HNSCC. Moreover, we noticed that EZR could affect the immunotherapy and specific drug sensitivity, making it an underlying clinical target.

## 2 METHODS

### 2.1 Screening of NK cell marker genes

Data of RNA sequences and related clinical information of 528 HNSC patients were gathered from the TCGA database (accessed time: 2023/7/11, https://tcga‐data.nci.nih.gov/tcga/), and the high‐throughput sequencing data were transformed into TPM values.^14^ Based on multiple immune infiltration algorithms including CIBERSORT, EPIC, MCPCOUNTER, QUANTISEQ, TIMER and XCELL algorithms from the online tool (www.home‐for‐researchers.com), values of NK cells for each patient were quantified individually.^15^, ^16^, ^17^, ^18^, ^19^, ^20^ After observing the distribution of the data, EPIC, QUANTISEQ and XCELL algorithms were screened for the differential expression analysis, and the co‐expressed genes including upregulated and downregulated genes were also retained as the NK cell‐related genes.

### 2.2 Related single‐cell analysis and prognostic analysis

First, correlation analysis was performed on these NK cell‐related genes including SSNA1, TRIR, PAXX, DPP7, WDR34, EZR, PHLDA1 and ELOVL1. Then, differentially expressed genes (DEGs) analysis and univariate prognostic analysis were also conducted, followed by single‐cell analysis of these genes using the online tool (http://tisch.comp‐genomics.org/home/).^21^ Lastly, Kaplan–Meier (KM) curves were analysed and differences in clinical parameters including age, gender, grade and stage were also analysed.

### 2.3 Functional enrichment analysis

Gene set variation analysis (GSVA) was utilized to identify the potential molecular mechanisms between high‐ and low‐expression groups based on the Hallmark gene set from the molecular signature database (https://www.gsea‐msigdb.org/gsea/msigdb).^22^ Gene ontology (GO) enrichment analysis was performed to explore the different biological processes (BP) and molecular functions (MF) of the differently expressed genes between two groups.^23^

### 2.4 Genomic feature analysis

Differential analyses of tumour mutation burden (TMB) and tumour stemness index (mRNAsi) were performed to examine the genomic landscape of molecules involved in two groups. With the input of “SNP6” files, we calculated arm‐ and focal‐level somatic copy number alterations (SCNAs) in the tumour using GISTIC2, which were downloaded from the genomic data commons data portal (https://portal.gdc.cancer.gov/).^24^

### 2.5 Immune cell infiltration analysis

Three scores including the immune score, the stromal score and the estimate immune score calculated based on the “estimate” R package were used for the evaluation of immune cells infiltration. Furthermore, immunocyte infiltration analysis according to the 42‐immunocytes project was also performed between these two groups.

### 2.6 ICB therapy response and drug sensitivity prediction

The underlying ICB (PD‐L1, CTLA4, HAVCR2, LAG3 and PD1) response for HNSC patients was predicted with tumour immune dysfunction and exclusion (TIDE) analysis.^25^ Besides, the submap algorithm was also performed to reveal which group of patients may respond to anti‐PD‐1 and anti‐CTLA4 therapy. The potential chemotherapy response of the chemotherapy drugs for patients was evaluated using the Genomics of Drug Sensitivity in Cancer (GDSC) database.^26^

### 2.7 Statistical analysis

A version of R software 4.2.2 was used to perform all the R packages. Comparisons between two groups were analysed with Wilcoxon rank‐sum tests, and continuous variables were analysed using Wilcoxon rank‐sum tests. In all cases, *p* values were set up as two‐sided, and *p* values less than 0.05 were considered statistically significant.

## 3 RESULTS

### 3.1 Identification of NK cell marker genes

**FIGURE 1:**
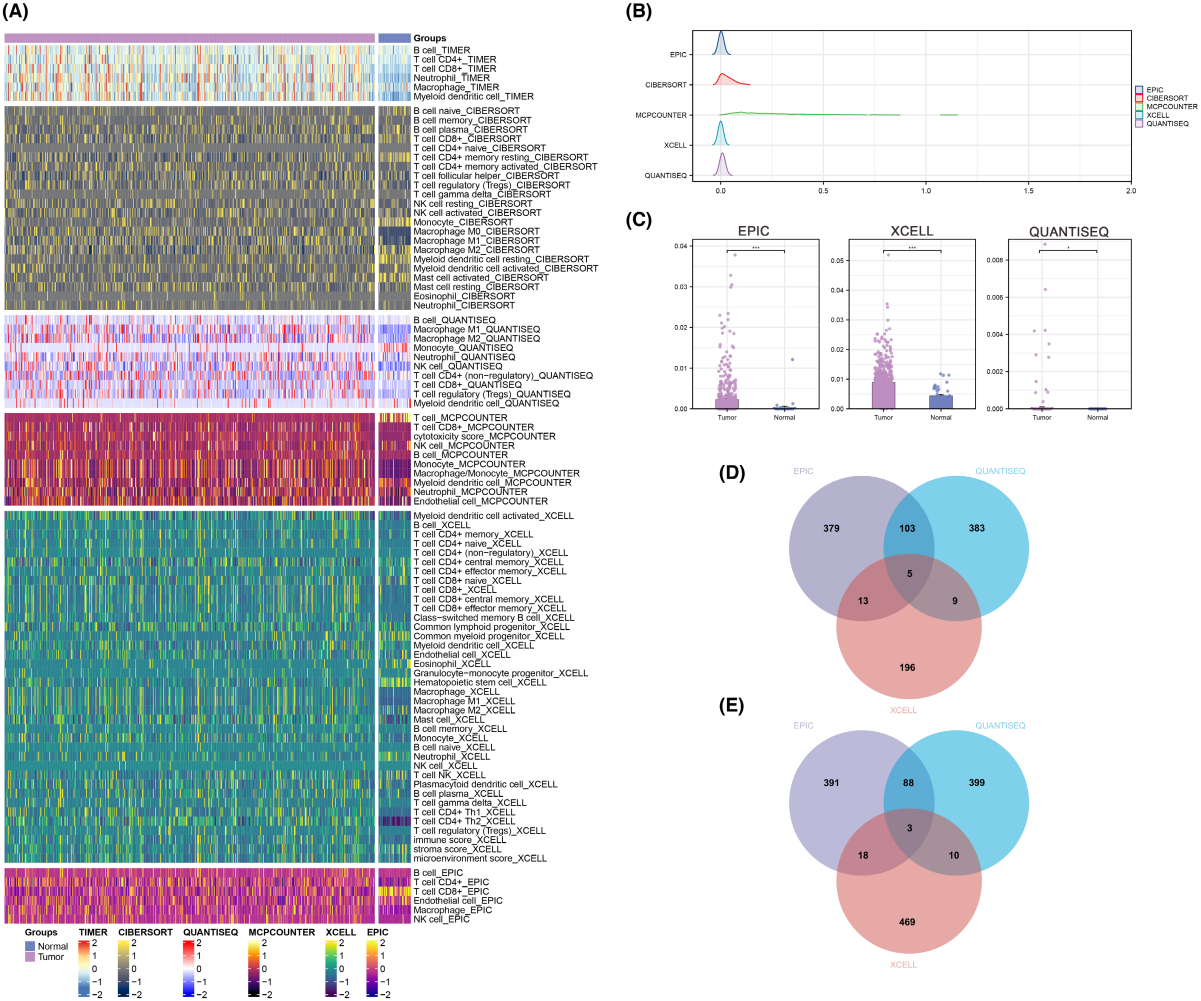
Identification of genes encoding markers for NK cells. (A) Quantitative analysis of the distribution of NK cells in each patient according to the CIBERSORT, EPIC, MCPCOUNTER, QUANTISEQ, TIMER, and XCELL algorithms. (B) Data distribution of the level of NK cell abundance. (C) Differential expression analysis of NK cell abundance in EPIC, XCELL and QUANTISEQ algorithms. (D,E) Wayne diagram of the differential up‐regulated (D) and down‐regulated (E) genes NK cell genes among three algorithms.

The whole flow chart of our study was shown in Figure S1. The heatmap of Figure 1A was generated to visualize the overall distribution of the level of NK cells quantified by CIBERSORT, EPIC, MCPCOUNTER, QUANTISEQ, TIMER and XCELL algorithms in each patient. According to our data, we found that the level of NK cell abundance in EPIC, QUANTISEQ and XCELL algorithms was relatively high (Figure 1B). Differential expression analysis revealed a notable difference in NK cell abundance between tumour and normal samples (Figure 1C). According to the Venn diagram, five common up‐regulated genes (Figure 1D) and three common down‐regulated genes (Figure 1E) were identified across these three algorithms.

### 3.2 Relationship between these genes and determination of the hub NK cell marker gene

**FIGURE 2:**
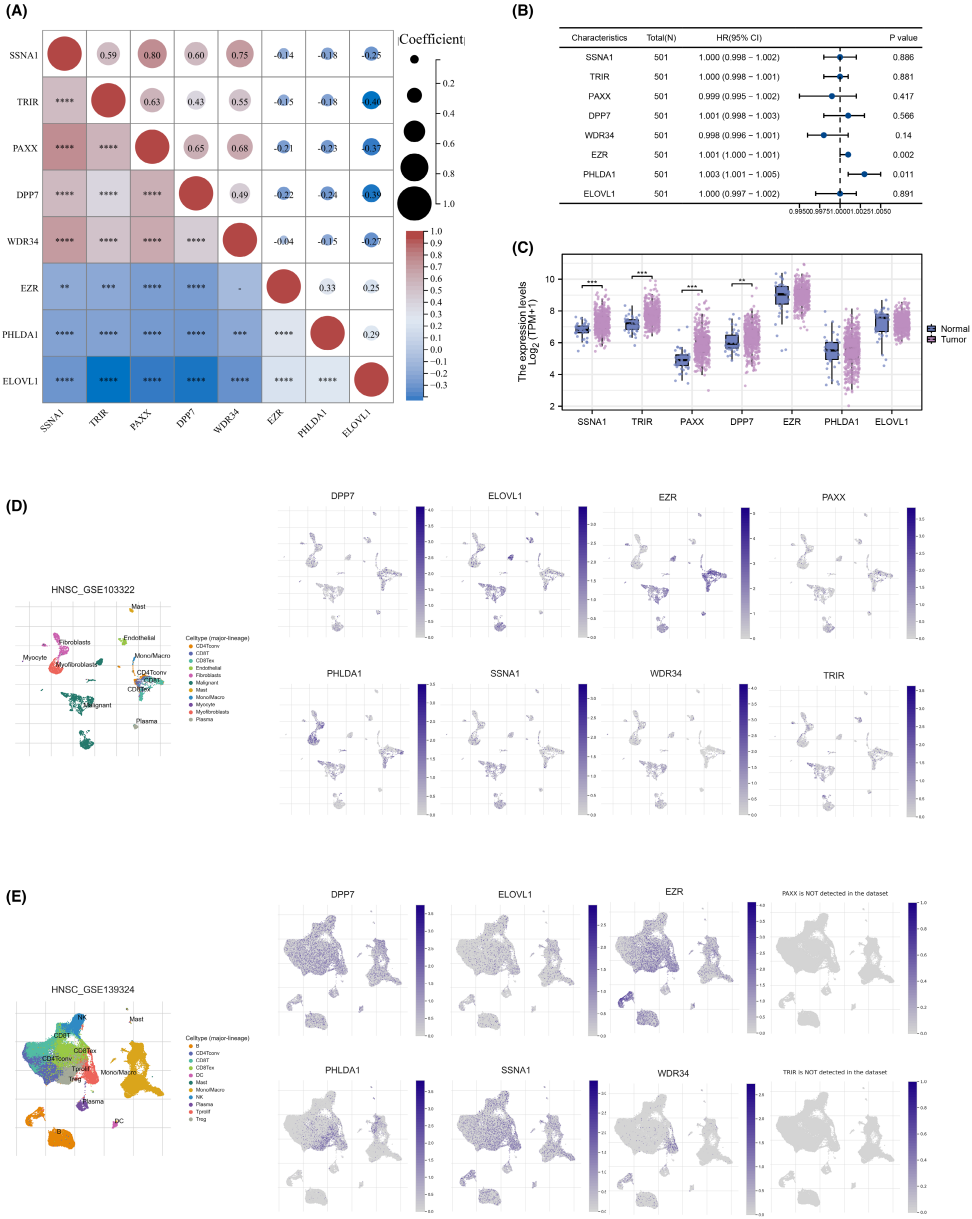
Analysis of the relationship between these genes and hub NK cell marker gene. (A) Correlation analyses between 8 genes associated with NK cell marker. (B) Univariate analysis of these genes and EZR was identified as the hub gene. (C) Differential expression analysis of these 8 NK cell marker genes. (D,E) Both GSE103322 and GSE139324 cohorts were used to analyse the expression distribution of these genes in multiple cell subgroups.

We first examined the pairwise correlation between genes using Pearson correlation analysis across HNSCC samples (Figure 2A). Univariate analysis was conducted to assess the prognostic significance of these genes, and EZR was identified for prognosis in HNSC patients with the lowest *p*‐value (Figure 2B). The differential expression was assessed for each gene and the EZR has the lowest *p* value (Figure 2C). Following this, the expression distribution of these genes in multiple cell subgroups was analysed both in the GSE103322 cohort and GSE139324 cohort (Figure 2D,E).

### 3.3 Analysis of clinical characteristics and functional enrichment analysis

**FIGURE 3:**
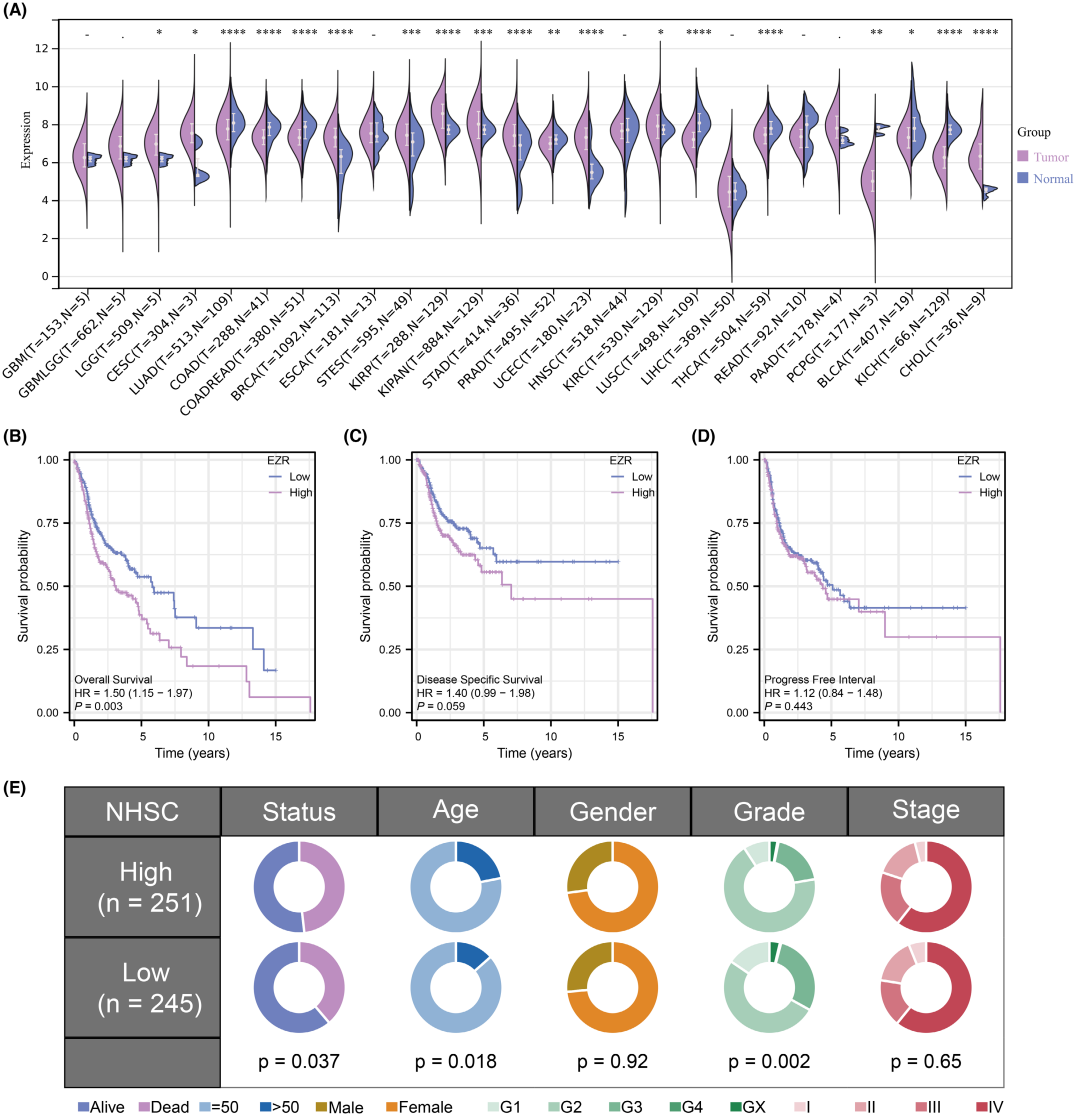
Clinical relevance analysis. (A) The levels of EZR expression among all tumours based on TCGA dataset. (B‐D) KM analyses of HNSC patients based on overall survival time, disease specific survival time and progress free interval time. (E) A comparison of two groups’ clinicopathological features including age, gender, grade and stage.

**FIGURE 4:**
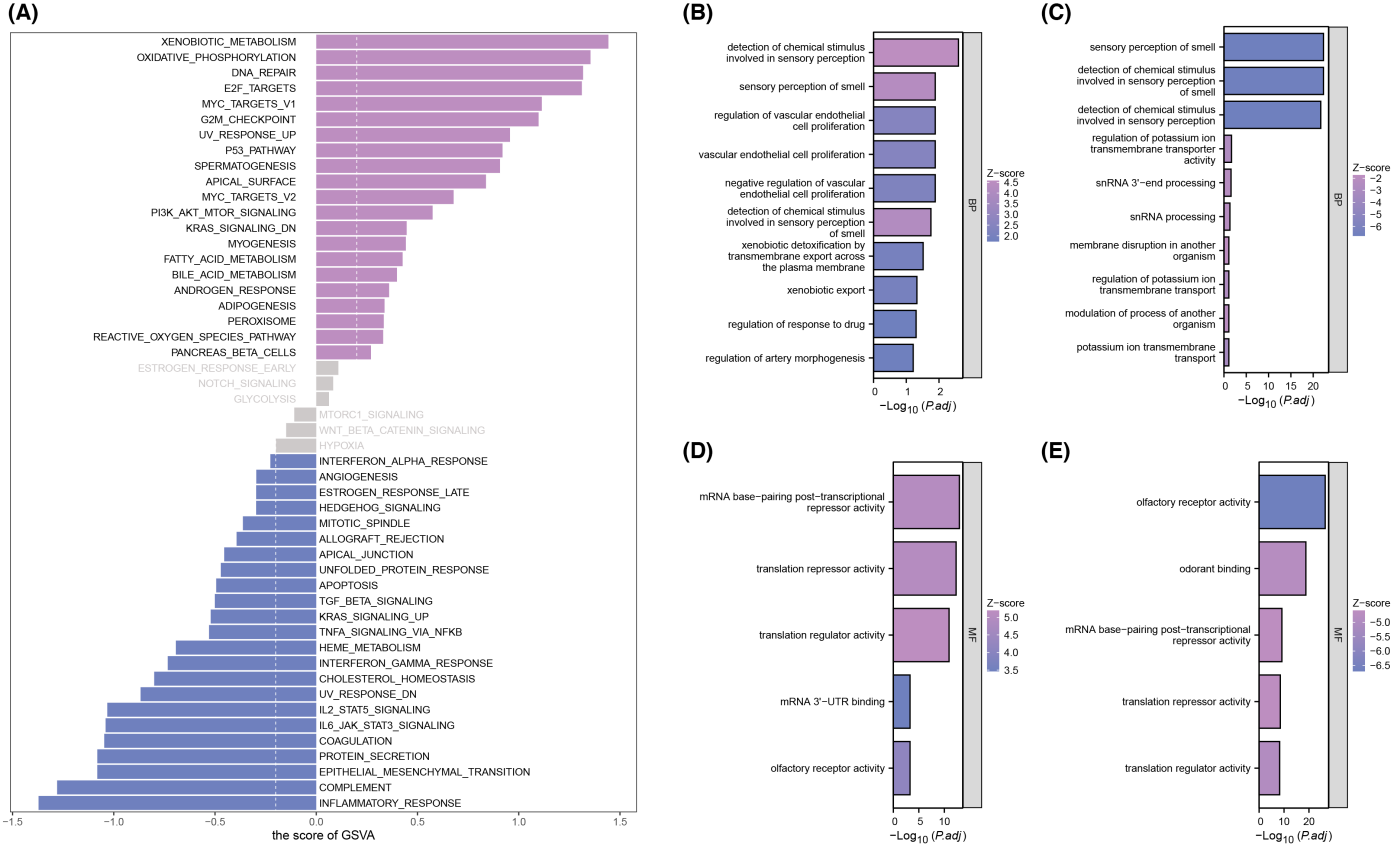
GSVA and GO enrichment analysis between two groups. (A) GSVA showing enriched pathways in two groups. (B,D) GO analysis showing BP and MF terms were enriched in high expression group. (C,E) GO analysis also revealed different BP and MF terms enriched in low expression group.

After prognostic analysis, EZR served as the key gene strongly associated with the prognosis of HNSCC patients. We found that EZR expressed highly in most tumours (Figure 3A). HNSCC patients were stratified into high‐ and low‐expression groups using the median value of EZR expression as a cutoff. KM survival curves based on overall survival, disease‐specific survival and progress‐free interval were all performed and a significant difference in overall survival time was observed between the two groups (Figure 3B–D). To unravel the profile of clinical manifestation for these two groups, clinical characteristics analysis was then performed (Figure 3E). Next, to explore the potential biological functions and signalling pathways between the two groups, GSVA analysis was conducted. In the high‐expression group, xenobiotic metabolism, oxidative phosphorylation, DNA repair and E2F targets were enriched. In the low‐expression group, inflammatory response, complement, epithelial‐mesenchymal transition (EMT) and protein secretion were enriched (Figure 4A). In terms of BP, regulation of vascular endothelial cell proliferation and regulation of drug response were enriched in the high expression group while the sensory perception of smell was enriched in the low expression group (Figure 4B,C). Besides, the enriched MF terms in the high expression group were mRNA base‐pairing post‐transcriptional repressor activity, translation repressor activity and translation regulator activity. The enriched MF terms in the low expression group were olfactory receptor activity, odorant binding, and mRNA base‐pairing post‐transcriptional repressor activity (Figure 4D,E).

### 3.4 Comparison of genomic characteristics between groups

**FIGURE 5:**
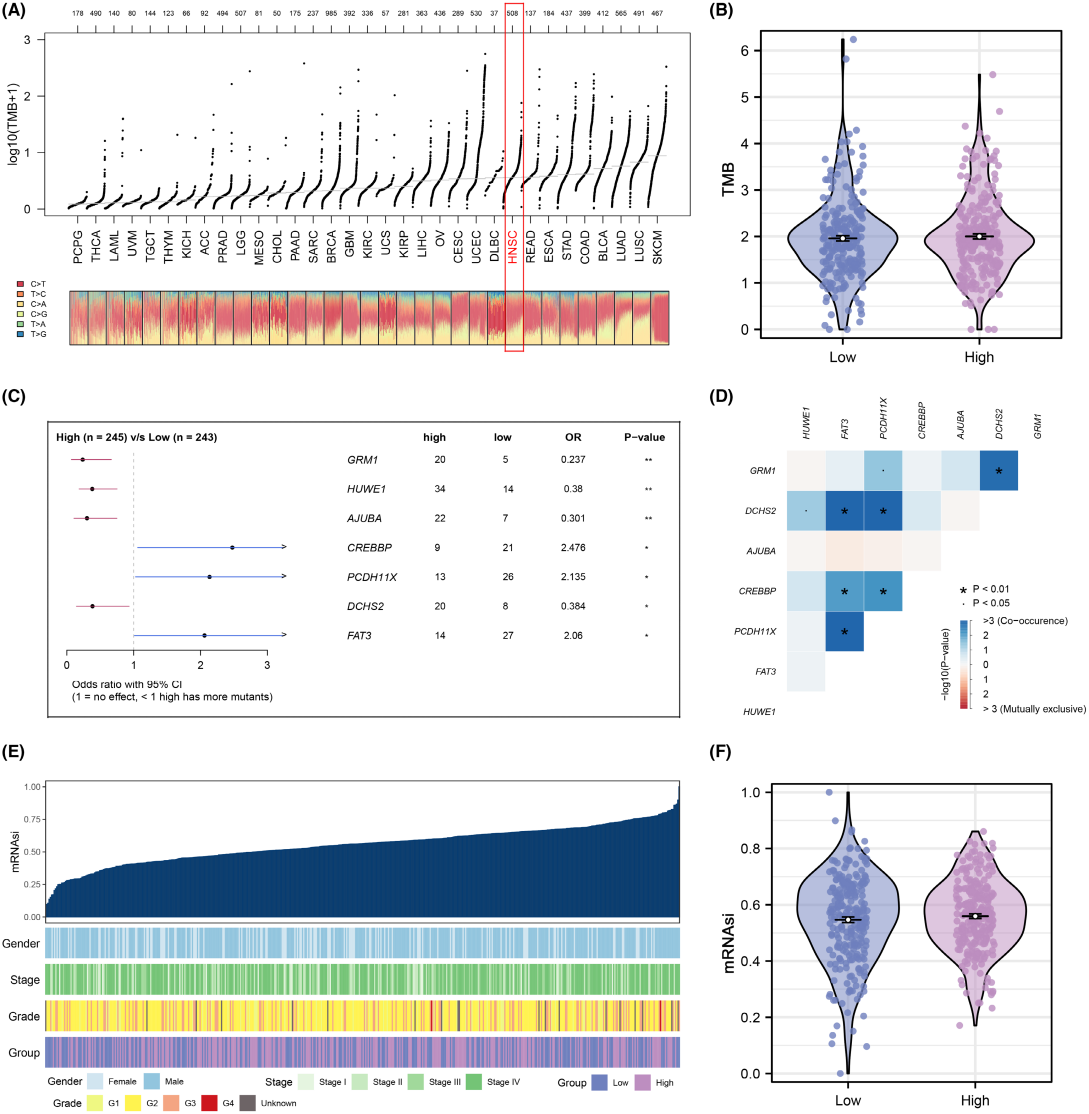
Analysis of differential genomic differences between two groups. (A) The levels of TMB value among all tumours based on TCGA dataset. (B) Differential expression analysis of TMB level between two groups in HNSC patients. (C) Identification of differential mutant genes between two group. (D) The relationship of co‐occurrence and exclusive between these differential mutant genes. (E) Data distribution of the level of mRNAsi score in each HNSC patient. (F) Differential expression analysis of mRNAsi score between two groups.

**FIGURE 6:**
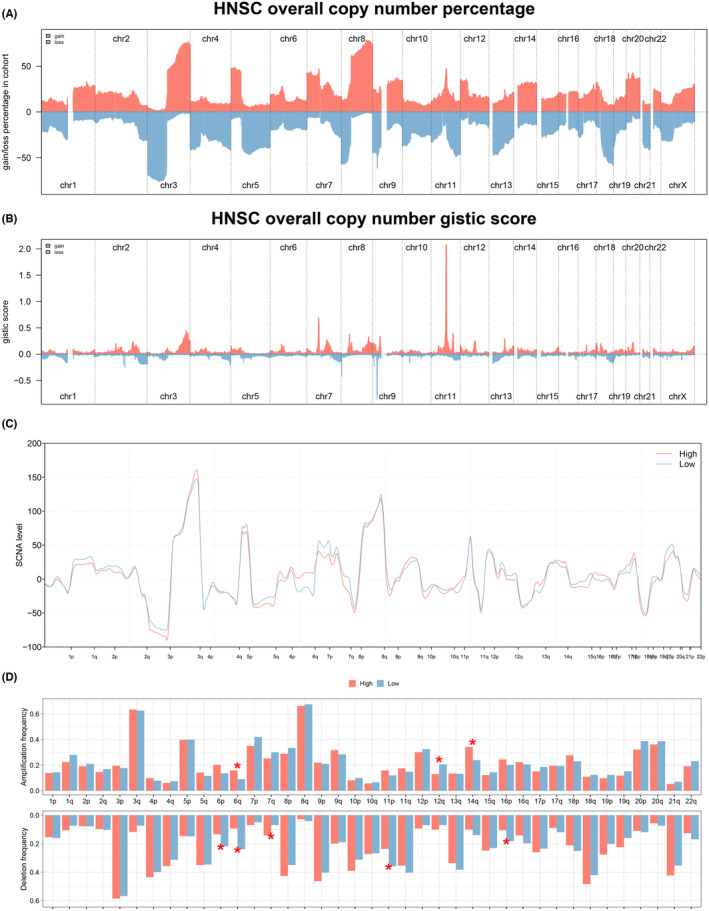
Analysis of chromosomal aberrations of between two groups. (A) The copy number percentage of each HNSC patient. (B) The copy number gistic score of each HNSC patient. (C,D) No significant difference of amplification and deletion frequencies was observed between two groups.

The TMB levels in the HNSCC cohort were relatively high in 33 different types of tumours (Figure 5A). However, in the HNSCC cohort, no significant difference in TMB level was detected between the two groups (Figure 5B). Then, genes associated with differential mutants (the number of mutations more than 20) were identified between two groups (Figure 5C), and the co‐occurrence and exclusive relationship between these differential mutant genes was shown in Figure 5D. In addition, an overview of the clinical characteristics and mRNAsi distribution for each sample can be found in Figure 5E, and no significant difference in mRNAsi level was observed from differential expression analysis (Figure 5F). Chromosomal aberrations of each HNSC patient including copy number percentage and copy number gistic score were assessed separately (Figure 6A,B). Also, we found that there is no significant difference in amplification and deletion frequencies between the two groups (Figure 6C,D).

### 3.5 Features of immune infiltration and prediction of ICB therapy

**FIGURE 7:**
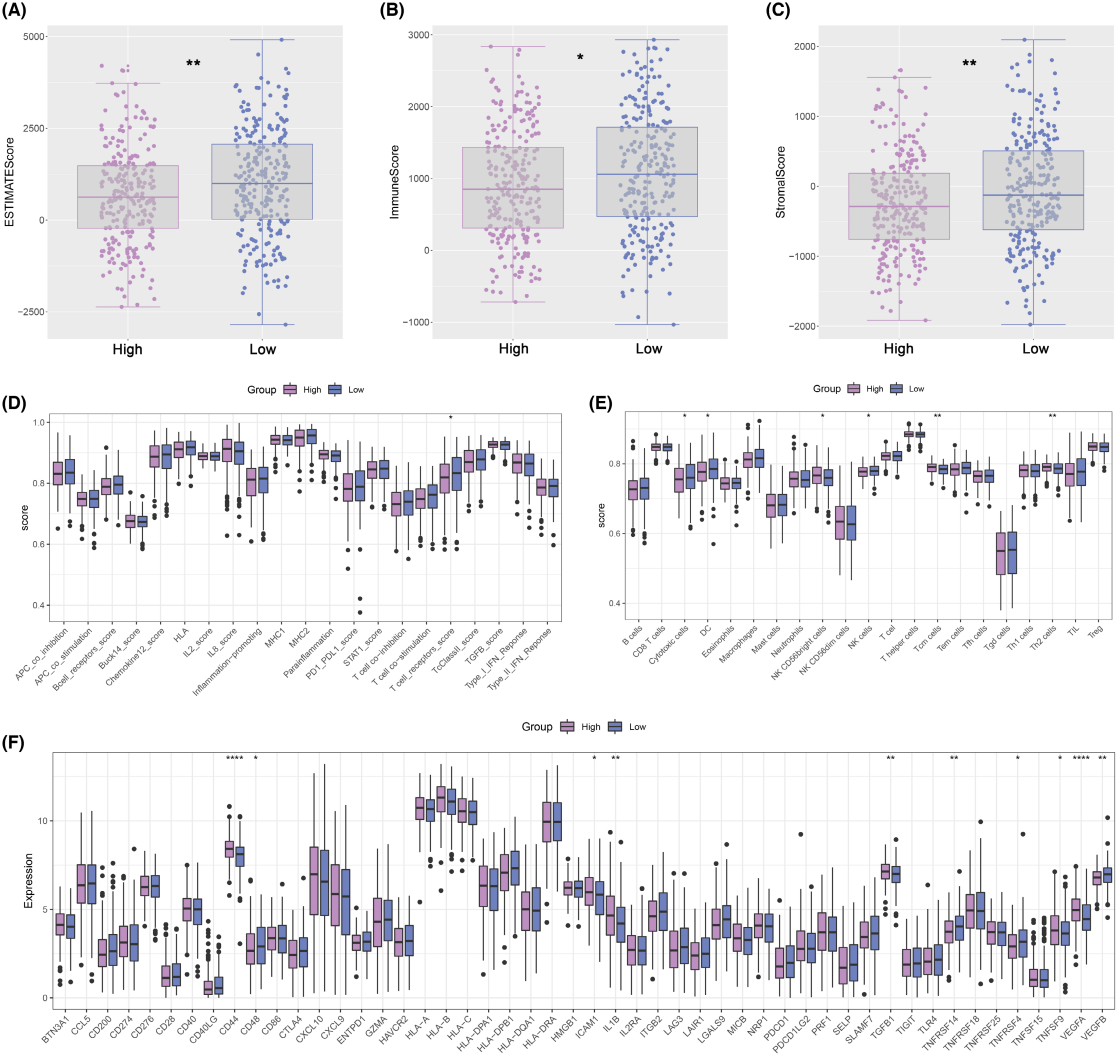
Analysis of immune cell infiltration landscape in HNSC patients. (A–C) Differential expression analysis of ESTIMATE, Immune, and Stroma scores between two groups. (D,E) The boxplots for the comparison of immune cells and immune functions between two groups. (F) The boxplots for the comparison of the immune checkpoints genes between two groups.

**FIGURE 8:**
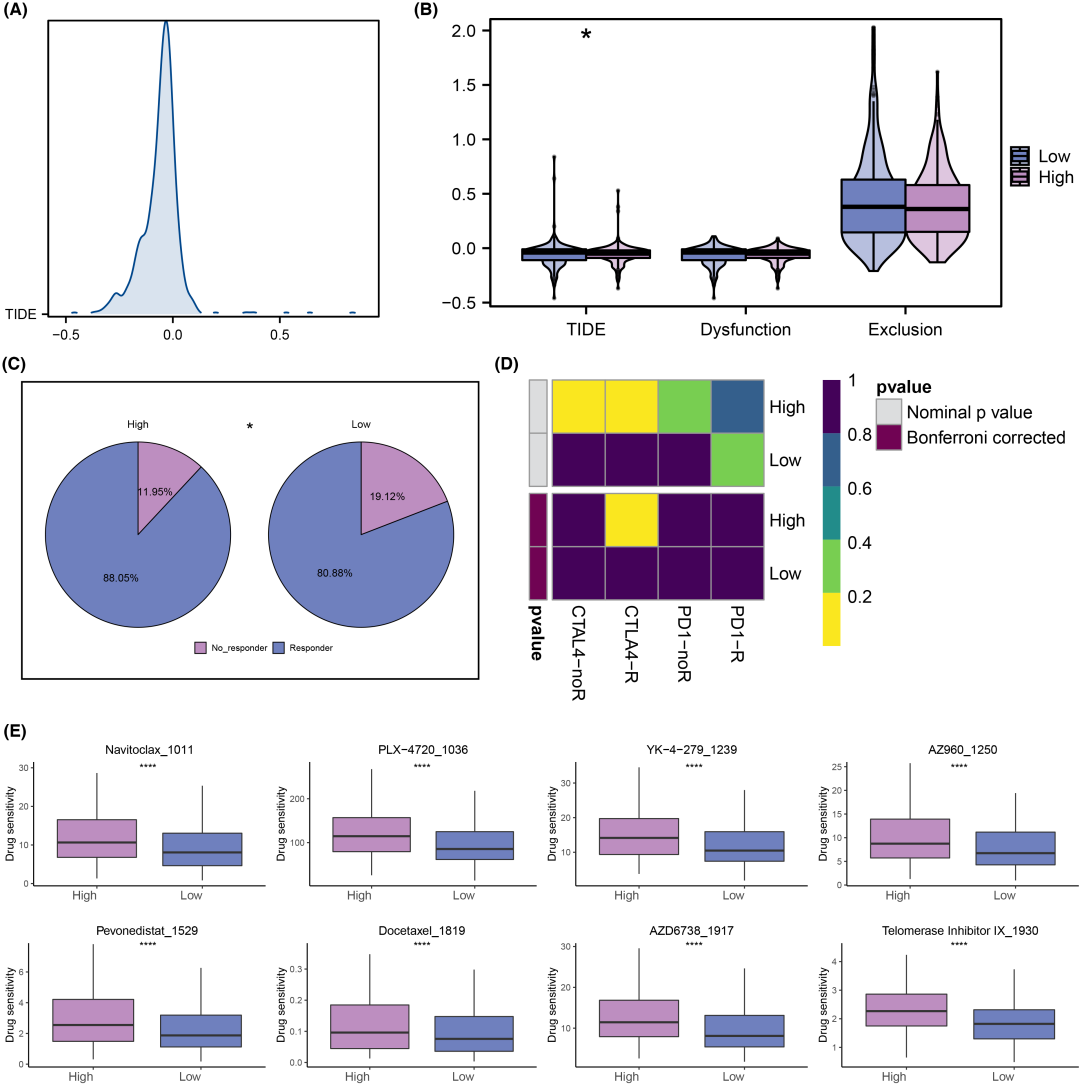
Prediction for immunotherapy response and identification of appropriate agents. (A) The distribution of TIDE scores in HNSC patients. (B) The comparison of TIDE, Dysfunction and Exclusion scores between two groups. (C) The proportion of responder and non‐responder to immunotherapy in two groups. (D) Submap analysis for two groups in HNSC also suggest that patients in high expression group would be respond to immunotherapy. (E) Box plots of estimated drug sensitivity for several chemotherapeutic agents in two groups.

According to ESTIMATE analysis, patients in the low‐expression group had higher immune, stromal and estimate scores than those in the high‐expression group (Figure 7A–C). Then, scores of immune function and immune cell abundance were calculated using the CIBERSORT and ssGSEA algorithms. The results showed that immune terms associated with the T‐cell were enriched in the high‐expression group (Figure 7D,E). Next, we compared the levels of immune checkpoint expression between the two groups and found that high‐expression patients were more highly expressed (Figure 7F). The distribution of TIDE scores was different among HNSCC patients (Figure 8A). The TIDE, disfunction and exclusion scores were lower in the high‐expression group (Figure 8B). According to TIDE analysis, patients in the high‐expression group may be more sensitive to ICB therapy (Figure 8C). Submap analysis also suggested that patients in the high‐expression group would tend to respond to anti‐CTLA4 therapy (Figure 8D). Finally, the drug sensitivity analysis revealed that patients in the high expression group may tend to respond to Navitoclax_1011, PLX − 4720_1036, YK − 4 − 279_1239, AZ960_1250, Pevonedistat_1529, Docetaxel_1819, AZD6738_1917 and Telomerase Inhibitor IX_1930 (Figure 8E).

## 4 DISCUSSION

HNSCC is the most common head and neck tumour with relatively high morbidity and mortality worldwide.^27^ Tobacco, alcohol intake and HPV infection are the main risk factors. Despite great advances in surgery, radiotherapy and chemotherapy techniques over the past few decades, the treatment of HNSCC still faces many challenges, especially for patients with advanced or recurrent disease.^28^ In recent years, targeted therapy has become a new direction in the treatment of HNSCC.^29^ By targeting specific biomarkers or signalling pathways, targeted drugs aim to reduce damage to normal cells, thereby improving treatment outcomes and patients' quality of life. However, further research is needed on its long‐term effects and safety.

In our study, we quantified the immune microenvironment of HNSCC through multiple algorithms. Next, we identified NK cell‐associated genes by quantifying NK cells, including SSNA1, TRIR, PAXX, DPP7, WDR34, EZR, PHLDA1 and ELOVL1. Then, we explored the single‐cell expression pattern of these genes in the HNSCC microenvironment. Univariate Cox regression analysis indicated that the EZR, PHLDA1 and ELOVL1 were related to the prognosis of HNSCC patients. Following this, we selected EZR for further analysis. Our results showed that the patients with high EZR expression might have a poor prognosis and worse clinical features. Biological enrichment analysis showed that EZR is associated with many oncogenic pathways and a higher tumour stemness index. Meanwhile, we found that EZR can remodel the immune microenvironment of HNSCC. Moreover, we noticed that EZR could affect the immunotherapy and specific drug sensitivity, making it an underlying clinical target.

Our results showed that the patients with high EZR expression might have a higher activity of xenobiotic metabolism, oxidative phosphorylation, G2M checkpoint, PI3K/AKT/mTOR signalling, fatty acid metabolism and bile acid metabolism. Oxidative phosphorylation is the main energy‐producing process of the cell, involving the generation of ATP.^30^ In cancer, the role of oxidative phosphorylation is complex. While many cancer cells prefer glycolysis for energy production due to the Warburg effect, some tumour cells still rely on or enhance oxidative phosphorylation.^31^ Enhanced oxidative phosphorylation provides cancer cells with the necessary energy and biosynthesis to support their rapid proliferation and growth.^32^ In specific cases, enhanced oxidative phosphorylation in cancer cells is associated with chemotherapy resistance and cancer stem cell properties. The G2M checkpoint is a critical stage in the cell cycle responsible for ensuring that DNA has been replicated intact before cells enter mitosis.^33^ During cancer development, this checkpoint is often disturbed. Cancer cells may bypass the impaired G2M checkpoint, causing them to continue dividing even in the presence of DNA damage, which further leads to the accumulation of genetic mutations.^34^ The PI3K/AKT/mTOR signalling pathway plays a key role in many biological processes, including cell growth, proliferation, metabolism and survival.^35^ In cancer, this pathway is often activated to promote tumour formation and progression. Abnormal PI3K activity can lead to overactivation of AKT, which in turn activates mTOR, which promotes protein synthesis, cell proliferation and inhibits apoptosis. In addition, this pathway is also closely related to cancer drug resistance, metastasis and cancer stem cell properties.^36^ The role of lipid metabolism in cancer has received extensive attention in recent years.^37^ Cancer cells often reprogram their lipid metabolism pathways to meet their demands for rapid proliferation and growth. This not only provides the cancer cell with a source of energy but also with the necessary components of the cell membrane and signalling molecules.^38^ Our results provide indications for the mechanism of action of EZR in HNSCC.

Meanwhile, we found that EZR was associated with a higher tumour stemness index and can remodel the tumour microenvironment of HNSCC. Cancer stem cells are a class of cells with self‐renewal and multilineage differentiation capabilities and are regarded as key factors in cancer development, recurrence and metastasis.^39^ Cancer stem cells play a crucial role in HNSCC. These cells exhibit strong resistance to traditional treatments such as chemotherapy and radiotherapy, making the treatment of HNSCC a great challenge.^40^ Therefore, in‐depth understanding and targeting of cancer stem cells in HNSCC have become the focus of current research. The tumour microenvironment refers to the environment composed of cells and matrix around tumour cells, including immune cells, fibroblasts, vascular cells, and extracellular matrix.^41^ This environment plays a critical role in cancer progression, immune evasion, and therapy resistance. In HNSCC, the role of the tumour microenvironment is particularly pronounced. The tumour microenvironment of HNSCC is often accompanied by chronic inflammation, which promotes tumour development and metastasis.^42^ In addition, cells in the microenvironment can promote resistance of HNSCC cells to treatment.

Although our analysis is based on high‐quality data and code, some limitations still need to be noted. Firstly, due to the systematic bias of bioinformatics itself, the results of its analysis cannot fully reflect the true situation within the organism. Secondly, potential biases between samples still affect the stability of the conclusion, such as lineage bias. Finally, although we identified EZR as the research gene, further experimental validation is still needed.

## AUTHOR CONTRIBUTIONS

**Peng Shao:** Conceptualization (equal); data curation (equal); investigation (equal); resources (equal); validation (equal); visualization (equal). **Wei‐Wei Hu:** Conceptualization (equal); formal analysis (equal); methodology (equal); resources (equal); software (equal); supervision (equal). **Xin‐lian Shi:** Conceptualization (equal); formal analysis (equal); methodology (equal); resources (equal); software (equal). **Ming‐yang Shu:** Conceptualization (equal); data curation (equal); resources (equal); software (equal). **Dong‐Ya Li:** Conceptualization (equal); methodology (equal); software (equal). **Tingting Zhou:** Conceptualization (equal); funding acquisition (equal); resources (equal); software (equal). **Qi‐tao Zhao:** Conceptualization (equal); data curation (equal); formal analysis (equal); supervision (equal); validation (equal).

## FUNDING INFORMATION

None.

## CONFLICT OF INTEREST STATEMENT

None.

## Supporting information


Figure S1.

## Data Availability

All data are available from the corresponding author upon reasonable request.

## References

[1] Johnson DE , Burtness B , Leemans CR , Lui VWY , Bauman JE , Grandis JR . Head and neck squamous cell carcinoma. Nat Rev Dis Primers. 2020;6(1):92. 33243986 10.1038/s41572-020-00224-3 PMC7944998

[2] Kitamura N , Sento S , Yoshizawa Y , Sasabe E , Kudo Y , Yamamoto T . Current trends and future prospects of molecular targeted therapy in head and neck squamous cell carcinoma. Int J Mol Sci. 2020;22(1):240. 33383632 10.3390/ijms22010240 PMC7795499

[3] Brakenhoff RH , Wagner S , Klussmann JP . Molecular patterns and biology of HPV‐associated HNSCC. Recent Results Cancer Res. 2017;206:37‐56. 27699528 10.1007/978-3-319-43580-0_3

[4] Cramer JD , Burtness B , Le QT , Ferris RL . The changing therapeutic landscape of head and neck cancer. Nat Rev Clin Oncol. 2019;16(11):669‐683. 31189965 10.1038/s41571-019-0227-z

[5] Du E , Mazul AL , Farquhar D , et al. Long‐term survival in head and neck cancer: impact of site, stage, smoking, and human papillomavirus status. Laryngoscope. 2019;129(11):2506‐2513. 30637762 10.1002/lary.27807 PMC6907689

[6] von Witzleben A , Wang C , Laban S , Savelyeva N , Ottensmeier CH . HNSCC: tumour antigens and their targeting by immunotherapy. Cell. 2020;9(9):2013. 10.3390/cells9092103 PMC7564543 32942747

[7] Masarwy R , Kampel L , Horowitz G , Gutfeld O , Muhanna N . Neoadjuvant PD‐1/PD‐L1 inhibitors for Resectable head and neck cancer: a systematic review and meta‐analysis. JAMA Otolaryngol Head Neck Surg. 2021;147(10):871‐878. 34473219 10.1001/jamaoto.2021.2191 PMC8414366

[8] Wu SY , Fu T , Jiang YZ , Shao ZM . Natural killer cells in cancer biology and therapy. Mol Cancer. 2020;19(1):120. 32762681 10.1186/s12943-020-01238-x PMC7409673

[9] Crinier A , Narni‐Mancinelli E , Ugolini S , Vivier E . SnapShot: natural killer cells. Cell. 2020;180(6):1280. 32200803 10.1016/j.cell.2020.02.029

[10] Terrén I , Orrantia A , Vitallé J , Zenarruzabeitia O , Borrego F . NK cell metabolism and tumor microenvironment. Front Immunol. 2019;10:2278. 31616440 10.3389/fimmu.2019.02278 PMC6769035

[11] Jacobs B , Ullrich E . The interaction of NK cells and dendritic cells in the tumor environment: how to enforce NK cell & DC action under immunosuppressive conditions? Curr Med Chem. 2012;19(12):1771‐1779. 22414086 10.2174/092986712800099857

[12] Xie G , Dong H , Liang Y , Ham JD , Rizwan R , Chen J . CAR‐NK cells: a promising cellular immunotherapy for cancer. EBioMedicine. 2020;59:102975. 32853984 10.1016/j.ebiom.2020.102975 PMC7452675

[13] Sivori S , Pende D , Quatrini L , et al. NK cells and ILCs in tumor immunotherapy. Mol Aspects Med. 2021;80:100870. 32800530 10.1016/j.mam.2020.100870

[14] Wang Z , Jensen MA , Zenklusen JC . A practical guide to the cancer genome atlas (TCGA). Methods Mol Biol. 2016;1418:111‐141. 27008012 10.1007/978-1-4939-3578-9_6

[15] Chen B , Khodadoust MS , Liu CL , Newman AM , Alizadeh AA . Profiling tumor infiltrating immune cells with CIBERSORT. Methods Mol Biol. 2018;1711:243‐259. 29344893 10.1007/978-1-4939-7493-1_12 PMC5895181

[16] Racle J , Gfeller D . EPIC: a tool to estimate the proportions of different cell types from bulk gene expression data. Methods Mol Biol. 2020;2120:233‐248. 32124324 10.1007/978-1-0716-0327-7_17

[17] Becht E , Giraldo NA , Lacroix L , et al. Estimating the population abundance of tissue‐infiltrating immune and stromal cell populations using gene expression. Genome Biol. 2016;17(1):218. 27765066 10.1186/s13059-016-1070-5 PMC5073889

[18] Plattner C , Finotello F , Rieder D . Deconvoluting tumor‐infiltrating immune cells from RNA‐seq data using quanTIseq. Methods Enzymol. 2020;636:261‐285. 32178821 10.1016/bs.mie.2019.05.056

[19] Li T , Fan J , Wang B , et al. TIMER: a web server for comprehensive analysis of tumor‐infiltrating immune cells. Cancer Res. 2017;77(21):e108‐e110. 29092952 10.1158/0008-5472.CAN-17-0307 PMC6042652

[20] Aran D , Hu Z , Butte AJ . xCell: digitally portraying the tissue cellular heterogeneity landscape. Genome Biol. 2017;18(1):220. 29141660 10.1186/s13059-017-1349-1 PMC5688663

[21] Sun D , Wang J , Han Y , et al. TISCH: a comprehensive web resource enabling interactive single‐cell transcriptome visualization of tumor microenvironment. Nucleic Acids Res. 2021;49(D1):D1420‐d1430. 33179754 10.1093/nar/gkaa1020 PMC7778907

[22] Hänzelmann S , Castelo R , Guinney J . GSVA: gene set variation analysis for microarray and RNA‐seq data. BMC Bioinformatics. 2013;14:7. 23323831 10.1186/1471-2105-14-7 PMC3618321

[23] Yu G , Wang LG , Han Y , He QY . clusterProfiler: an R package for comparing biological themes among gene clusters. Omics. 2012;16(5):284‐287. 22455463 10.1089/omi.2011.0118 PMC3339379

[24] Mermel CH , Schumacher SE , Hill B , Meyerson ML , Beroukhim R , Getz G . GISTIC2.0 facilitates sensitive and confident localization of the targets of focal somatic copy‐number alteration in human cancers. Genome Biol. 2011;12(4):R41. 21527027 10.1186/gb-2011-12-4-r41 PMC3218867

[25] Fu J , Li K , Zhang W , et al. Large‐scale public data reuse to model immunotherapy response and resistance. Genome Med. 2020;12(1):21. 32102694 10.1186/s13073-020-0721-z PMC7045518

[26] Yang W , Soares J , Greninger P , et al. Genomics of drug sensitivity in cancer (GDSC): a resource for therapeutic biomarker discovery in cancer cells. Nucleic Acids Res. 2013;41(Database issue):D955‐D961. 23180760 10.1093/nar/gks1111 PMC3531057

[27] Chen SMY , Krinsky AL , Woolaver RA , Wang X , Chen Z , Wang JH . Tumor immune microenvironment in head and neck cancers. Mol Carcinog. 2020;59(7):766‐774. 32017286 10.1002/mc.23162 PMC7282929

[28] Rassy E , Nicolai P , Pavlidis N . Comprehensive management of HPV‐related squamous cell carcinoma of the head and neck of unknown primary. Head Neck. 2019;41(10):3700‐3711. 31301162 10.1002/hed.25858

[29] Alsahafi E , Begg K , Amelio I , et al. Clinical update on head and neck cancer: molecular biology and ongoing challenges. Cell Death Dis. 2019;10(8):540. 31308358 10.1038/s41419-019-1769-9 PMC6629629

[30] Lehninger AL , Wadkins CL . Oxidative phosphorylation. Annu Rev Biochem. 1962;31:47‐78. 14463786 10.1146/annurev.bi.31.070162.000403

[31] Ashton TM , McKenna WG , Kunz‐Schughart LA , Higgins GS . Oxidative phosphorylation as an emerging target in cancer therapy. Clin Cancer Res. 2018;24(11):2482‐2490. 29420223 10.1158/1078-0432.CCR-17-3070

[32] Vander Heiden MG , Cantley LC , Thompson CB . Understanding the Warburg effect: the metabolic requirements of cell proliferation. Science. 2009;324(5930):1029‐1033. 19460998 10.1126/science.1160809 PMC2849637

[33] Pedroza‐Garcia JA , Xiang Y , De Veylder L . Cell cycle checkpoint control in response to DNA damage by environmental stresses. Plant J. 2022;109(3):490‐507. 34741364 10.1111/tpj.15567

[34] Ciardo D , Goldar A , Marheineke K . On the interplay of the DNA replication program and the intra‐S phase checkpoint pathway. Genes. 2019;10(2):94. 30700024 10.3390/genes10020094 PMC6410103

[35] Xia P , Xu XY . PI3K/Akt/mTOR signaling pathway in cancer stem cells: from basic research to clinical application. Am J Cancer Res. 2015;5(5):1602‐1609. 26175931 PMC4497429

[36] Guerrero‐Zotano A , Mayer IA , Arteaga CL . PI3K/AKT/mTOR: role in breast cancer progression, drug resistance, and treatment. Cancer Metastasis Rev. 2016;35(4):515‐524. 27896521 10.1007/s10555-016-9637-x

[37] Bian X , Liu R , Meng Y , Xing D , Xu D , Lu Z . Lipid metabolism and cancer. J Exp Med. 2021;218(1):e20201606. 33601415 10.1084/jem.20201606 PMC7754673

[38] Li D , Li Y . The interaction between ferroptosis and lipid metabolism in cancer. Signal Transduct Target Ther. 2020;5(1):108. 32606298 10.1038/s41392-020-00216-5 PMC7327075

[39] Prasetyanti PR , Medema JP . Intra‐tumor heterogeneity from a cancer stem cell perspective. Mol Cancer. 2017;16(1):41. 28209166 10.1186/s12943-017-0600-4 PMC5314464

[40] Huang T , Song X , Xu D , et al. Stem cell programs in cancer initiation, progression, and therapy resistance. Theranostics. 2020;10(19):8721‐8743. 32754274 10.7150/thno.41648 PMC7392012

[41] Elmusrati A , Wang J , Wang CY . Tumor microenvironment and immune evasion in head and neck squamous cell carcinoma. Int J Oral Sci. 2021;13(1):24. 34341329 10.1038/s41368-021-00131-7 PMC8329257

[42] Strait AA , Wang XJ . The role of transforming growth factor‐beta in immune suppression and chronic inflammation of squamous cell carcinomas. Mol Carcinog. 2020;59(7):745‐753. 32301180 10.1002/mc.23196 PMC7282943

