# Supplementary figures and images for: Exploring NK‐Cell molecules that impact the immune response and microenvironment in head and neck squamous cell carcinoma
Source: J Cell Mol Med. 2023 Nov 27;28(2):e18045. doi: 10.1111/jcmm.18045 (PMC10826436; doi:10.1111/jcmm.18045)

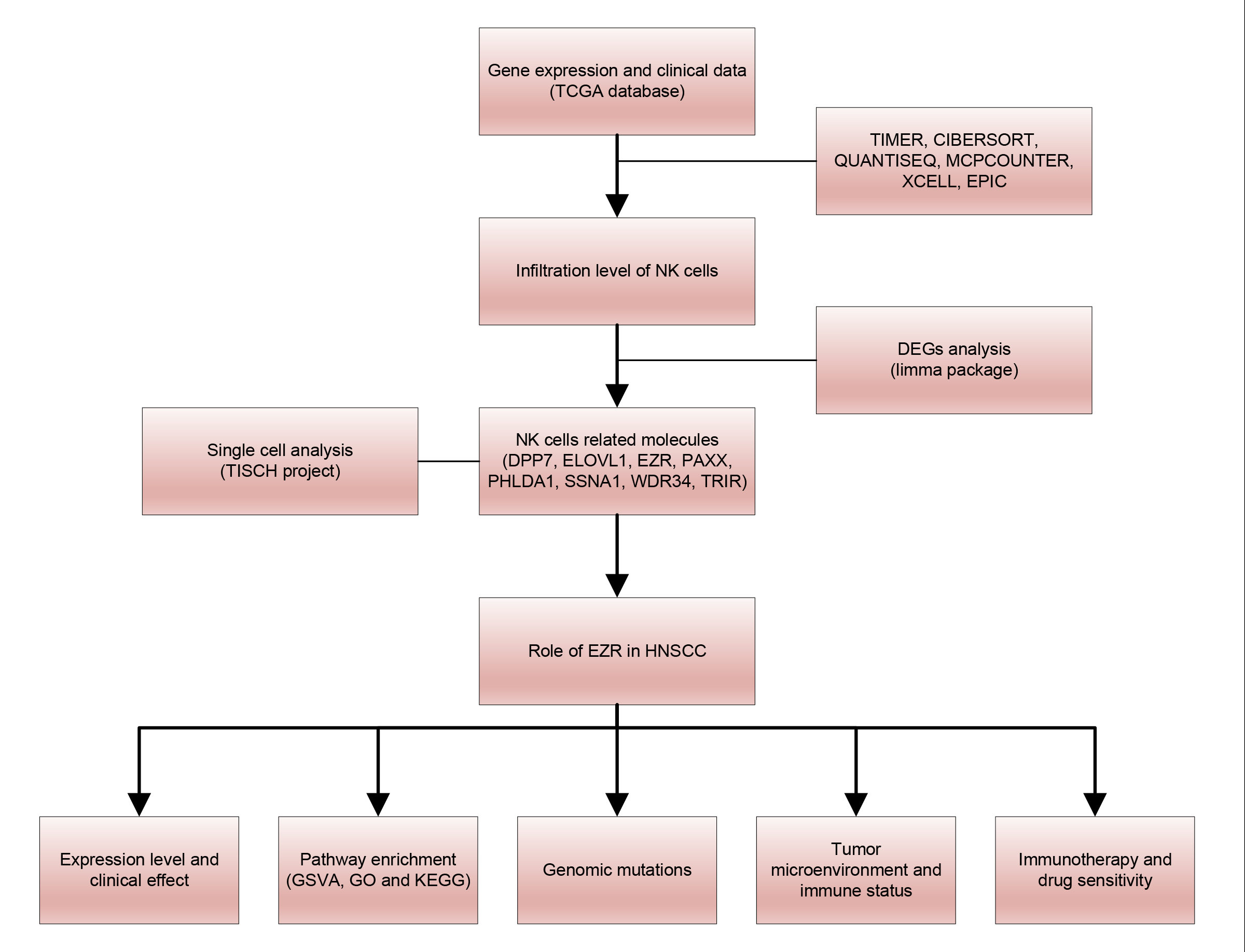

Supplement: Supplementary file 1 — Figure S1. [file JCMM-28-e18045-s001.tif]
